# What Else Is Needed to Improve Survival from Out-of-Hospital Cardiac Arrest to Hospital Admission? Data from a Prospective Registry for the Years 2020–2023 in the Italian Province of Varese

**DOI:** 10.3390/jcm12237264

**Published:** 2023-11-23

**Authors:** Roberto De Ponti, Carlo Arnò, Andrea Piemonti, Paola Centineo, Paola Genoni, Michele Golino, Simone Savastano, Guido Garzena, Sabina Campi

**Affiliations:** 1 Department of Medicine and Surgery, University of Insubria-Varese, Cardiology Unit, Ospedale di Circolo, ASST Settelaghi, 21100 Varese, Italymgolino@uninsubria.it (M.G.); 2 Agenzia Regionale Emergenza Urgenza, Articolazione Aziendale Territoriale—118 Varese, 21100 Varese, Italysabina.campi@asst-settelaghi.it (S.C.); 3 Cardiology Unit, IRCCS Fondazione Policlinico S. Matteo, 27100 Pavia, Italy; s.savastano@smatteo.pv.it; 4 Agenzia Tutela della Salute Insubria, 21100 Varese, Italy

**Keywords:** out-of-hospital cardiac arrest, ventricular fibrillation, sudden cardiac death, COVID-19 pandemic

## Abstract

Around the world, data on out-of-hospital cardiac arrest (OHCA) are heterogeneous in terms of outcomes and reporting, and not all registries follow the Utstein recommendations for uniform OHCA data collection. This study reports data on OHCA occurring in recent years in a limited territory to analyze, in a homogenous setting, the circumstances and interventions affecting survival to hospital admission. OHCA data from the province of Varese for the years 2020–2022 were extracted from a prospective registry. For survival to hospital admission, the impact of pandemic waves and variables known to affect survival was evaluated both in the overall population and in the subgroup of patients in whom cardiopulmonary resuscitation (CPR) was initiated or continued by the emergency medical service (EMS). Overall, 3263 OHCAs occurred mainly at home (88%), with a time to intervention of 13.7 min, which was significantly longer during lockdown (15.7 min). Bystanders performed CPR in 22% of the cases and used automatic external defibrillator (AED) in 2.2% of the cases. Overall survival to hospital admission was 7.7%. In the multivariate analysis, in the general population, occurrence near a public building (OR 1.92), the presence of witnesses (OR 2.65), and a shockable rhythm (OR 7.04) were independent predictors of survival to hospital admission, whereas age (OR 0.97) and occurrence during a pandemic wave (OR 0.62) were associated with significantly worse survival to hospital admission. In the group of patients who received CPR, AED shock by bystanders was the only independent predictor of survival (OR 3.14) to hospital admission. Among other factors, early defibrillation was of crucial importance to improve survival to hospital admission in possibly rescuable patients. The occurrence of OHCA during pandemic waves was associated with longer intervention time and worse survival to hospital admission.

## 1. Introduction

Out-of-hospital cardiac arrest (OHCA) represents a major public health problem worldwide, affecting hundreds of thousands of patients each year with a generally poor outcome in terms of both survival and neurological outcome [1]. To improve the prognosis of patients suffering from OHCA, the so-called chain of survival has been implemented: it defines the necessary steps from early recognition and calls for help to post-resuscitation care, with a particular emphasis on early cardiopulmonary resuscitation (CPR) and early defibrillation [2]. Despite many efforts, after decades, the survival rate remains strikingly low. This prompted the European Society of Cardiology to include in the 2022 guidelines, a class I recommendation for an improvement in public access to defibrillators, the promotion of CPR by bystanders, and the training of the general population in basic life support [3]. Furthermore, there is a paucity of data on OHCA and heterogeneity in reporting [4,5], even in the same nation [4]. In addition, the coronavirus disease (COVID-19) pandemic has become an important additional variable affecting the public health system with a strong impact on OHCA [6]. All these factors taken together greatly limit the identification of the priorities for improvements in OHCA management.

This study reports data on OHCAs that occurred in the province of Varese in the years 2020–2022, which were extracted from the Lombardia CARe prospective registry to analyze the variables affecting survival to hospital admission in this territory. Although the data from the province of Varese represent approximately one-fifth of the entire registry, the aim is to analyze the activity of a single dispatch center to obtain data as homogeneous as possible to identify corrective actions to improve survival to hospital admission in this specific setting.

## 2. Materials and Methods

### 2.1. Study Population and Settings of the Emergency Medical System

The population reported in this study is extracted from the Lombardia Cardiac Arrest Registry (Lombardia CARe: NCT03197142) for the OHCAs that occurred only in the province of Varese. As reported in detail elsewhere [7], the Lombardia CARe is a multicenter longitudinal prospective registry, which currently enrolls all OHCAs occurring in the following provinces of the Lombardia region: Pavia, Lodi, Cremona, Mantua, Brescia, Como, and Varese. All data are collected according to the 2014 Utstein recommendations, which is the appropriate way to collect and report data on OHCA to uniform the characteristics of OHCA registries [8]. In this region, emergency medical services (EMSs) are provided and coordinated by the “Agenzia Regionale Emergenza Urgenza (AREU)”, which ensures standardization of the emergency healthcare procedures and data collection for every rescue event. Data regarding each OHCA are subsequently centralized electronically in the coordinating center located in the Department of Cardiology of the Fondazione IRCSS Policlinico San Matteo, Pavia, Italy. All data are checked by the coordinating center in Pavia for incongruencies and inaccuracies, which are communicated to the local centers and corrected accordingly.

For the present study, OHCAs occurring between 1 January 2020, when the local agency of AREU joined the registry, and 31 December 2022 are included. The province of Varese is in the north-western part of the Lombardia region and has an area of 1198 km^2^ with a population of 877,668 permanent residents, as assessed on 1 January 2023. The mean age of permanent residents is 46.6 years, and the mean life expectancy is 81.1 and 85.5 years for men and women, respectively. Mountains and rural regions are in the northern part of the province, urban areas are in the southern part, and the western part is characterized by lakes and rivers. In this territory, the official number of public automatic external defibrillators (AEDs) is 1859 [9], largely underestimated considering the ones in sports centers, where their presence is mandatory by law, and in private buildings. The public AEDs distributed in the territory are kept in sealed alarmed cases that can be reached and opened by anybody at any time. The location of the nearest AED can be communicated by a dispatcher to a caller or can be found on the application “DAEdove”, which can be freely downloaded on a mobile device. Every time an AED is used, this information is notified automatically to the dispatching center. In this region, a system including professional first responders, such as police officers and firefighters, is not present nor is a text message system to alert trained volunteers. The rescue calls for the province of Varese are handled by a single dispatch center in the northern part of the region, which coordinates ambulances staffed with personnel trained in basic life support and defibrillation (BLS-D), vehicles for advanced life support equipped also with a mechanical device for CPR (Easy Pulse, Schiller, Baar, Switzerland), and one helicopter staffed with a physician and a specialized nurse. In case of a suspected OHCA, the dispatcher activates one to three emergency vehicles with at least one physician and offers assistance to the calling bystander to guide the first resuscitation maneuvers, which is defined as phone-guided CPR. BLS-D-trained personnel from the EMS are instructed to start resuscitation unless clear signs of death are present (rigor mortis, hypostasis, and injuries not compatible with life), while the duration of resuscitation attempts is left to the physician. CPR included only chest compressions both in case it was performed by pre-trained personnel or it was phone-guided. The patients were then transferred to the nearest hospital.

This study was approved by the Ethical Committee of Insubria (study number 138/2019) and followed the approval of the coordinating center in 2015. Data were anonymized. Informed consent was obtained from survivors whenever possible, especially for their neurologic conditions. In all the other cases, which represent the vast majority, given that the global survival to hospital discharge was lower than 10%, according to the protocol approved by the Ethical Committees, it was considered legitimate to waive informed consent, as this is the only possible way to conduct this type of study, which collects data with the aim of improving survival from OHCA. Moreover, data on patients declared dead in the field were collected anonymously.

### 2.2. Data Management and Analysis

Data are collected and managed using Research Electronic Data Capture (REDCap version 13.1.33), a secure, web-based software platform, hosted at Fondazione IRCCS Policlinico San Matteo, Pavia, Italy. After each event, the personnel of the EMS completes a relevant electronic report, which is automatically transferred to the coordinating center. After the initial rescue, the surviving patients are admitted to the hospitals in the area coordinated by the local EMS, and the data are collected by the local hospital investigators and evaluated for data quality by the Lombardia CARe Study Management Team.

For this study, together with patient gender and age, the following variables were extracted from the database: time to intervention, defined as the time elapsed from the telephone call by bystanders to the dispatch center and the arrival of the first rescue team; site of the OHCA; whether the OHCA was witnessed, defined as such only in case that bystanders directly witnessed the patient collapse; CPR initiated by bystanders, both independently or phone-guided; patients declared dead upon EMS personnel arrival; the use of AED by bystanders; the presenting rhythm at AED classified as shockable or non-shockable; and the occurrence of recovery of spontaneous circulation (ROSC), if any, before or after hospital admission. The evaluated outcome was survival to hospital admission, which was evaluated as survival to hospital admission in the overall population, survival to hospital admission in the group of patients in whom resuscitation maneuvers were initiated or continued by EMS, and survival to hospital admission in the Utstein comparator group [8]. This last group consists of witnessed OHCAs with a shockable rhythm, and it is generally used to assess the efficiency of the EMS.

According to the GPS location coordinates, the site of an OHCA was classified as at “home” if it was located at the patient’s home address, in “buildings” if it was located in public-access buildings or factories, where an AED was close by, and “other” if it was located in rural areas, mountains, or on the shores of rivers or lakes. To evaluate the relationship with the COVID-19 pandemic, a sub-analysis of OHCA occurrence during the pandemic waves or during the timeframes between two pandemic waves was carried out. The five established waves of the pandemic in Italy were identified as follows [10]: first wave, from March to May 2020; second wave, from October 2020 to January 2021; third wave, from February to May 2021; fourth wave, from June to October 2021; and fifth wave, from November 2021 to February 2022.

### 2.3. Statistics

Categorical variables are presented as counts and percentages, and continuous variables are presented as median and the 25th–75th interquartile range (IQR). A chi-squared test was used for the comparison of categorical variables. The Kruskal–Wallis test was used to compare skewed continuous variables. Binary logistic regression analyses, both unadjusted and adjusted, were used to assess the association between independent factors and survival to hospital admission. Results are reported as Odds Ratios (ORs) with 95% confidence intervals (CIs), which were graphed using Forest plots. Since in a preliminary analysis, a non-negligible proportion of cases were declared already dead upon arrival of EMS personnel and, consequently, no resuscitation maneuvers were initiated, two univariate and multivariate logistic regression analyses were performed. The first was performed on the overall population of cases and the second on the population of cases in which resuscitation maneuvers were continued or initiated by EMS personnel. The first analysis aimed at evaluating the impact on survival to hospital admission of the circumstances in which the OHCA occurred, while the second evaluated the impact on survival to hospital admission of the interventions undertaken on a patient considered rescuable. Therefore, in addition to basic patient demographic data, in the first analysis, the following variables were considered: time to intervention, site of OHCA and if witnessed, presenting rhythm at AED, and occurrence during a pandemic wave. In the second analysis, the site of OHCA, time to intervention, CPR by bystanders, phone-guided CPR, and AED shock by bystanders were the included variables. The OR for the continuous variables refers to the probability of survival to hospital admission for each additional time unit (year for age and minute for time to intervention). Statistical analyses were performed using R statistical software version 4.3.0 (The R Foundation). The significance level was set at 0.05.

## 3. Results

### 3.1. OHCAs Occurring in the Years 2020–2022 in the Province of Varese

In the considered time frame, 3263 OHCAs occurred in a population with a median age of 78 years [IQR 66–86], predominantly of the male gender (59%). Table 1 reports data related to the OHCA events with a per-year breakdown. The considered variables are stable over time with no statistically significant difference across the three considered years. Notably, most of the events occurred at home, only 43% of the OHCAs were witnessed, and 35% of the cases were declared dead upon the EMS personnel’s arrival. Heart rhythm was detected using an AED in 74% of the entire population, and it was shockable in only 12% of the cases. CPR maneuvers were initiated by bystanders in 723 cases (22%), and it was performed under phone guidance in 432 cases (60%). In 193 cases, phone-guided CPR was proposed by the dispatch center but not accepted by bystanders. AED was minimally used by bystanders: in only 72 cases (2.2%), it was connected to the patient, and the shock was delivered in an even smaller group of patients, totaling only 23 and accounting for only 32% of the cases in which the AED was used by bystanders. Importantly, the bystander interventions were comparable in the three considered years with no significant variation over time.

Table 2 shows the relevant characteristics of the group of patients in whom CPR was initiated or continued by EMS personnel and of the group of patients declared dead upon the EMS personnel’s arrival. Between the two groups, there was no significant difference in age and the relationship with pandemic waves, while the first group experienced more frequently witnessed OHCAs and had a significantly higher prevalence of CPR by bystanders, shorter time to intervention, and fewer occurrences at home.

Table 3 reports the comparison between the patients who had a shockable rhythm and those who exhibited a non-shockable rhythm at AED. Patients exhibiting a shockable rhythm were significantly younger and of the female gender, had a shorter time to intervention, and were more likely to suffer from witnessed OHCAs outside of the home.

### 3.2. Time to Intervention According to the Year, Pandemic Wave, and Site of OHCA

In the overall population, the time to intervention was 13.7 (10.9–17) min. Table 4 shows the time to intervention according to the year, pandemic wave, and site of OHCA. There is a clear reduction over the years, with the shortest time to intervention observed in the year 2022, compared with the two previous years taken together: 13 (10–16) vs. 14 (11.2–17.5) minutes (*p* < 0.001). This variation was clearly related to the COVID-19 pandemic. In fact, the shortest times to intervention were observed during both the fourth and fifth waves, which had a milder impact on the EMS. A similarly short time was observed between the pandemic waves, while the longest was recorded during the second wave, which had the biggest impact on the local health system. Consequently, the time to intervention was significantly longer during the 2020 lockdown (first and second waves) compared with the other waves and the time periods between waves taken together (15.7 (12.5–19) vs. 13.1 (10.6–16.3) min; *p* < 0.001). Finally, OHCAs occurring at home had a significantly longer time to intervention compared with the other locations, although the difference was less marked (13.9 (11–17) vs. 13 (10–17); *p* = 0.01).

### 3.3. Survival to Hospital Admission

As reported in Table 1, 252 patients (7.7%) survived to hospital admission. ROSC was obtained before hospital admission in 232 patients (92%), and the remaining 20 patients had ROSC in hospital. Of the 3263 OHCAs, 230 (7%) were directly witnessed by the EMS. Of these, 63 (27%) had a ROSC either before hospitalization or once at the hospital. A mechanical compressor was used by the EMS in 234 of the 2120 (11%) cases who received CPR, of whom 44 (19%) survived to hospital admission. Survival to hospital admission was stable with no significant variation over the years.

Table 5 reports the distribution of the considered variables in the groups of survivors and non-survivors to hospital admission. Survivors were significantly younger with no gender difference between the two groups. As expected, in the survivor group, the percentage of witnessed OHCA, occurrence outside the home, presence of shockable rhythm, bystander-initiated CPR, and bystanders’ use of AED were significantly higher. In the survivor group, the rate of OHCAs occurring during the pandemic waves was significantly lower and, in this group, a significantly shorter time to intervention was observed.

Moreover, if only patients for whom CPR was initiated or continued by EMS personnel are considered, the overall rate of survival to hospital admission increases to 12% (252/2120), being identical in the three considered years. Finally, in the Utstein Comparator Group including only witnessed OHCA with a shockable rhythm, the rate of survival to hospital admission increases to 38%, as 76 patients survived in the group of 198 patients fulfilling these criteria.

### 3.4. Variables Affecting Survival to Hospital Admission

**Figure 1:**
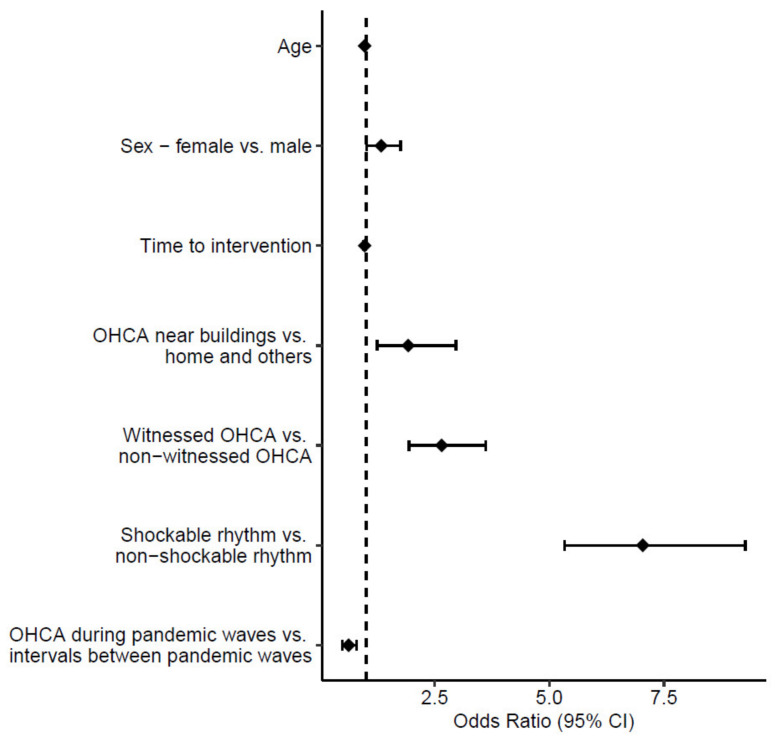
Forest plot presenting the result of the multivariate logistic regression analysis of survival to hospital admission in the overall population of 3263 patients.

In the univariate analysis on the general population (Table 6), only patient gender was not significant. Among the others, the site of OHCA occurrence, a witnessed OHCA, and a shockable rhythm showed higher ORs (3.00 (95% CI 2.09–4.32), 6.30 (95% CI 4.73–8.40), and 10.36 (95% CI 8.10–13.30), respectively). The multivariate analysis confirmed that these three variables were independent predictors of survival to hospital admission with higher OR values (Figure 1), especially for witnessed OHCAs and the presence of a shockable rhythm (2.65 (95% CI 1.94–3.61) and 7.04 (95% CI 5.33–9.28), respectively). Moreover, in the multivariate analysis, the time to intervention reached only borderline significance with an OR of 0.97 (95% CI 0.95–0.99) and *p* = 0.05. Finally, the occurrence of an OHCA during a pandemic wave was associated with a reduced survival to hospital admission probability, as confirmed with the multivariate analysis, with an OR of 0.62 (95% CI 0.48–0.79) and *p* = 0.002.

**Figure 2:**
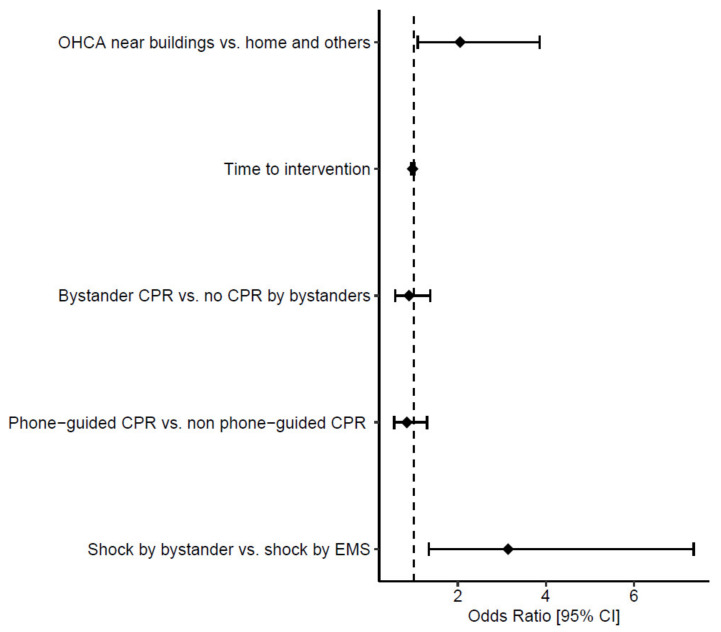
Forest plot presenting the result of the multivariate logistic regression analysis for survival to hospital admission in patients in whom CPR was initiated or continued by the emergency medical service.

In the population of 2120 patients for whom CPR was initiated or continued by EMS personnel (Table 7), all the considered variables were associated with significantly improved survival to hospital admission in the univariate analysis, with the occurrence of OHCA near buildings and shock delivered by bystanders showing the highest ORs (2.37 (95% CI 1.62–3.47) and 3.11 (95% CI 1.42–6.83), respectively). The comparison between CPR by bystanders and no CPR by bystanders evaluates patients who received CPR from bystanders vs. those who did not, where those who did not receive CPR by bystanders received CPR only lately by EMS personnel upon their arrival. In the multivariate analysis, shock delivery by bystanders before EMS arrival was the only independent predictor of survival to hospital admission, with an OR of 3.14 ((95% CI 1.34–7.36) *p* = 0.028), as shown in Figure 2.

## 4. Discussion

### 4.1. Main Findings

Our data on OHCA show a very stable scenario over time in terms of incidence, demographic characteristics of the population, circumstances in which the OHCA occurred, rhythm of presentation, use of CPR and AED by bystanders, and survival to hospital admission. In our study, OHCA had an incidence of approximately one case over 1000 inhabitants, occurred in a public place in 12% of the cases, and 35% of the patients were declared dead upon EMS arrival. The median time to intervention was 13.7 min, and the percentage of bystanders performing CPR and bystanders’ use of AED was as low as 22% and 2.2%, respectively, with a survival to hospital admission of 7.7%. Importantly, among patients for whom CPR was initiated or continued by the EMS, the only independent predictor of survival to hospital admission was early defibrillation by bystanders.

Most of these data are comparable to the ones of a recently published meta-analysis of 42 studies on OHCA in Italy published between 1995 and 2022 [4]. This meta-analysis showed low rates of bystander CPR and use of AED, which in our study is confirmed in a more homogeneous setting. This highlights the weakness of the initial ring of the chain of survival in OHCA management.

The COVID-19 pandemic had a clear detrimental impact on survival to hospital admission, as the OHCA occurrence during a pandemic wave was an independent predictor of death in the general OHCA population. The impact of pandemic waves on survival to hospital admission could have been mediated by several factors. Among others, the significantly longer time to intervention during pandemic waves, which is a well-known crucial factor in OHCA, could have played a major role. In fact, a significantly longer time to intervention was observed in the subgroup of patients declared dead upon the EMS personnel’s arrival and in those with a non-shockable rhythm at AED. The longer time to intervention reflects the fact that EMS resources were diverted toward the prevalent disease during the pandemic waves. In fact, a large Korean study on OHCA showed that EMS-related factors had the highest impact on outcomes during the pandemic [11]. Importantly, the time to intervention does not necessarily include the time between collapse and the phone call to the EMS, an uncontrollable variable that dramatically increases the time between OHCA occurrence and EMS arrival. It can be hypothesized that during the pandemic waves, the time before phone calls was significantly longer, reflecting the general attitude of the population to stay at home and avoid contact with the healthcare system for fear of contagion. This was associated with a striking increase in OHCA occurrence in 2020 as compared with the same period of the previous year, especially in areas of early lockdown [12,13,14].

### 4.2. Characteristics of OHCAs in the Province of Varese in the Years 2020–2022

Our data show a prevalence of 88% of OHCAs occurring at home, higher than the one previously reported, which may vary from 68 to 75% [15,16]. This value could have been influenced by the lockdown periods in the considered years. OHCA occurring at home had a longer time to intervention and were less likely to receive CPR, while the occurrence of OHCA near public buildings was an independent predictor of survival to hospital admission compared with those occurring at home or in rural areas. Occurrence of OHCA at home has been historically associated with poorer outcomes [15] depending in part on the fact that these patients may be sicker and older. However, even in this setting, outcomes can be improved if public health initiatives are undertaken to teach to potential bystanders CPR and the use of AED or how to reach it faster [15,16].

In 35% of our patients, CPR was not initiated or continued by EMS personnel, being declared dead upon their arrival. This proportion is similar to the one reported in other Italian studies [4,17] and the EuReCa TWO European study [1]. Several factors, such as age, location of event, CPR by bystanders, and EMS-witnessed events, are independent factors influencing physicians’ decision to initiate or continue resuscitation maneuvers, which can depend also on the physicians’ attitude [17]. Importantly, in our study, patients for whom CPR was not initiated or continued by EMS personnel were more likely to have a non-witnessed OHCA at home with a longer time to intervention compared with those who received CPR, whereas age was not a discriminant as it was similar in both groups.

In our study, only 12% of patients exhibited a shockable rhythm at AED, while in a meta-analysis of Italian studies, the proportion of these patients was almost twice as much, although with significant heterogeneity [4]. Patients with a non-shockable rhythm were significantly older, and the OHCA was less frequently witnessed with a longer time to intervention. As already mentioned, this time does not include the time between the collapse and the call to the dispatching center, which cannot be quantified but was possibly longer during the COVID-19 pandemic. All these factors combined in this specific population and these years could have contributed to faster degeneration of a fast ventricular tachycardia/ventricular fibrillation into a non-shockable rhythm.

### 4.3. Variables Affecting Survival to Hospital Admission

In our population, the overall survival to hospital admission was 7.7%, and this value increased to 12% if only the population of patients for whom CPR was initiated or continued by EMS is considered. If, as recommended [8], survival to hospital admission is evaluated in the Utstein comparator group represented by witnessed OHCAs with initial shockable rhythm, criteria met by only 6% of our population, survival to hospital admission increases to 38%. This value is in line with the data of other European studies [1,18], and it is an indicator of the efficacy of the EMS in the group of patients who have the highest probability of survival.

In our study, in the population of patients for whom CPR was continued or initiated by EMS, the only independent predictor of survival to hospital admission was AED shock delivered by bystanders. Notably, the presence of a shockable rhythm was as low as 12%, and it was inversely associated with a longer time to intervention observed especially during the pandemic waves. Moreover, in the general population, CPR by bystanders and bystanders’ use of AEDs was very low. These values are in line with those of a meta-analysis of Italian studies [4] but are lower than those reported by other European multicenter studies [1,19]. In these studies, CPR was initiated by bystanders in 58% of the cases [1], a shockable rhythm was observed in 20% of cases, on average, with a range between 11 and 37% [1], and early defibrillation before EMS intervention was delivered by bystanders in 39% of the cases [19].

Our data confirms that early defibrillation plays a key role and that EMSs are efficient, as shown by the rate of survival to hospital admission in the Utstein comparator group. However, there is a clear weakness in the initial ring of the chain of survival, specifically in the bystanders’ use of CPR and AED. In our study, bystanders’ use of CPR was not an independent predictor of survival to hospital admission, but it should be considered that it was performed under phone guidance in most cases (60%), probably without a bystanders’ specific training, and thus, it was probably of low quality. It should be also considered that no system of professional first responders or alerts using text messages is available in our region. Data from the German Resuscitation Registry [20] show that, although there was a striking increase in phone-guided CPR from 22 cases in the years 2006–2010 to 5229 in the years 2016–2020, no improvement in the evaluated outcome of OHCA was observed.

### 4.4. Actions to Improve Survival

Currently, there is a wide body of evidence that early defibrillation [19,21,22], population training [15,23], and retraining [24] in basic life support techniques, even in school children [25], are all major determinants of improved outcomes in patients experiencing OHCA. Dispatching trained volunteers able to intervene in a nearby OHCA event using innovative technologies [26,27,28,29,30] achieves similarly good results. The importance of basic life support training is highlighted by a French population-based study in which a higher level of population education in basic life support was of benefit, regardless of the extent of AED deployment [23]. Although the goal is to create a system saving lives, empowering especially the initial rings of the chain of survival, there could be challenges mainly related to the resources for basic life support training in a wide population, the attitude to react to an OHCA scenario, and a fear of legal consequences when CPR and AED are used by non-medical personnel. In July 2021, a new law was enacted in Italy aiming at improving the system and saving lives for OHCA [31]. Among others, one article is dedicated to legal protection for lay rescuers performing bystander CPR and defibrillation. As expected, our data show that this law had no immediate effect on OHCA treatment in our province, since systematic public initiatives and actions should be undertaken to significantly improve the rescuing scenario in OHCA. In the considered timeframe, an unexpected and adjunctive challenge was represented by the COVID-19 pandemic that affected survival by different factors, mainly the re-allotment of EMS to the prevalent disease and the patients’ reluctancy to contact medical services for the fear of contagion that might have delayed the first telephone call [13]. In the future, healthcare strategies, in terms of resources and messages given to the general population, should be carefully planned to avoid pandemic outbreaks affecting EMSs in time-dependent cardiovascular diseases [11].

### 4.5. Limitations

Most studies consider the outcomes of survival to hospital discharge or the longer follow-up available, as in-hospital events further decrease the survival rate in patients alive at hospital admission. In this prospective registry, survival at 30 days is included in data collection, but this item is complete and confirmed only for patients admitted to our hospital. Therefore, both data on survival at hospital discharge and on neurologic outcome for the overall population of OHCA in the province of Varese are missing and, consequently, their prevalence cannot be adequately calculated. Although we recognize the importance of these outcomes at a longer follow-up, this limitation does not significantly affect the value of our data, which shows the crucial role of early interventions by bystanders. These are expected to increase both the survival to hospital admission and the general conditions of survivors, which, in turn, also increases the survival rate at a longer follow-up.

## 5. Conclusions

After the COVID-19 pandemic, during which OHCA had a longer time to intervention and worse survival to hospital admission, early defibrillation by bystanders, among other well-known factors, needs improvement to increase survival in possibly rescuable patients. These data call for public actions to support programs to improve the early ring of the chain of survival, in the light of the new Italian law protecting lay rescuers during CPR and AED use. This is in line with the last European Society of Cardiology guidelines [3], which recommend community-based training in CPR and the use of AED.

## Figures and Tables

**Table 1:** Patient demographics and variables associated with OHCA.

	2020	2021	2022	Overall
OHCA (N, %)	1149 (35)	1034 (32)	1080 (33)	3263
Male gender (N, %)	668 (58)	593 (57)	660 (61)	1921 (59)
Age (median, IQR)	77 (66–86)	78 (65–86)	78 (66–87)	78 (66–86)
Site of OHCA (N, %)				
-Home	1003 (87)	911 (88)	954 (88)	2868 (88)
-Buildings	59 (5)	51 (5)	46 (4)	156 (5)
-Other	87 (8)	72 (7)	80 (8)	239 (7)
Witnessed (N, %)	477 (42)	469 (45)	458 (42)	1404 (43)
Dead on EMS arrival (N, %)	392 (34)	351 (34)	400 (37)	1143 (35)
Rhythm detected with AED (N, %)	894 (78)	771 (75)	760 (70)	2425 (74)
-Shockable (N, %)	107 (12)	94 (12)	99 (13)	300 (12) *
-Non-shockable (N, %)	787 (88)	677 (88)	661 (87)	2125 (88) *
CPR by bystanders (N, %)	245 (21)	228 (22)	250 (23)	723 (22)
Phone-guided CPR offered (N, %)	215 (19)	203 (20)	207 (19)	625 (19)
Use of EAD by bystanders (N, %)	28 (2)	17 (2)	27 (3)	72 (2.2)
Survivors to hospital admission (N, %)	91 (7.9)	80 (7.7)	81 (7.5)	252 (7.7)

* The percentage is given on the population of 2425 patients in whom a rhythm was detected. Abbreviations: AED = automatic external defibrillator; CPR = cardiopulmonary resuscitation; EMS = emergency medical service; OHCA = out-of-hospital cardiac arrest.

**Table 2:** Comparison between the two groups of patients who received CPR or were declared dead by emergency medical services.

	Patients Receiving CPR	Patients Declared Dead	*p*-Value
OHCA patients (N, %)	2120 (65)	1143 (35)	
Age (median, IQR)	79 (67–86)	78 (64.5–86)	0.169
Male gender (N, %)	841 (40)	479 (42)	0.317
OHCA at home (N, %)	1815 (86)	1053 (92)	<0.00001
Witnessed OHCA (N, %)	1220 (58)	184 (16)	<0.00001
Time to intervention (median, IQR)	13.4 (10.9–16.8)	14 (11–17.6)	0.001
During COVID-19 pandemic waves (N, %)	1241 (59)	664 (58)	0.529
CPR by bystanders (N, %)	670 (32)	53 (5)	<0.00001

Abbreviations: CPR = cardiopulmonary resuscitation; IQR= interquartile range; OHCA = out-of-hospital cardiac arrest.

**Table 3:** Comparison between the group of patients who had a shockable rhythm and those who exhibited a non-shockable rhythm at AED.

	Patients with Shockable Rhythm	Patients with Non-Shockable Rhythm	*p*-Value
OHCA patients (N, %)	300 (12)	2125 (88)	
Age (median, IQR)	69 (59–80)	80 (69–87)	<0.00001
Male gender (N, %)	75 (25)	895 (42)	<0.00001
OHCA at home (N, %)	228 (76)	1874 (88)	<0.00001
Witnessed OHCA (N, %)	239 (80)	1054 (50)	<0.00001
During COVID-19 pandemic waves (N, %)	170 (57)	1266 (60)	0.367
Time to intervention (median, IQR)	12.3 (10.2–16)	13.8 (11–17)	<0.00001

Abbreviations: IQR = interquartile range; OHCA = out-of-hospital cardiac arrest.

**Table 4:** Time to intervention according to time and site of OHCA.

	Time to Intervention in Minutes (IQR)
Year 2020	14.7 (11.3–18)
Year 2021	13.6 (11.1–16.9)
Year 2022	13 (10–16)
In the first wave	15.4 (12.1–19)
In the second wave	15.8 (12.7–19.1)
In the third wave	14.4 (12–17.8)
In the fourth wave	12.9 (10.5–15.4)
In the fifth wave	13.5 (11–16.4)
During time frames between waves	13.1 (10.6–16.3)
OHCA at home	13.9 (11–17)
OHCA in buildings	13 (10.6–17)
OHCA in other locations	12.8 (10–17)

Abbreviations: IQR = interquartile range; OHCA = out-of-hospital cardiac arrest.

**Table 5:** Characteristics of the survivors and non-survivors to hospital admission.

	Non-Survivors	Survivors	*p*
Number of patients	3011	252	
Age (median, IQR)	79 (67–87)	70 (57.5–80)	<0.001
Male gender (N, %)	1761 (58%)	160 (63%)	0.148
Site of OHCA (N, %)			
-home	2676 (89%)	192 (76%)	<0.001 *
-buildings	128 (4%)	28 (11%)	
-other	207 (7%)	32 (13%)	
Witnessed OHCA (N, %)	1209 (40%)	195 (77%)	<0.001
Time to intervention (median, IQR)	13.8 (11–17.2)	12.6 (10–16)	<0.001
During a COVID-19 pandemic wave (N, %)	1780 (59%)	125 (50%)	0.004
ROSC before hospital admission (N, %)	-	232 (92%)	-
Shockable rhythm (N, %)	183 (6%)	117 (46%)	<0.001
CPR by bystanders (N, %)	615 (20%)	108 (43%)	<0.001
Phone-guided CPR (N, %)	542 (18%)	83 (33%)	<0.001
AED used by bystanders (N, %)	57 (2%)	15 (6%)	<0.001

* vs. buildings and others combined together. Abbreviations: AED = automatic external defibrillator; CPR = cardiopulmonary resuscitation; OHCA = out-of-hospital cardiac arrest; ROSC = return of spontaneous circulation.

**Table 6:** Logistic regression analysis of survival to hospital admission in the overall population of 3263 patients.

Variables	Unadjusted		Adjusted	
	OR (95% CI)	*p*-Value	OR (95% CI)	*p*-Value
Age	0.97 (0.96–0.98)	<0.001	0.97 (0.96–0.98)	<0.001
Sex—female vs. male	0.82 (0.66–1.03)	0.148	1.33 (1.01–1.75)	0.09
Time to intervention	0.96 (0.94–0.98)	0.002	0.97 (0.95–0.99)	0.05
OHCA near buildings vs. home and other	3.00 (2.09–4.32)	<0.001	1.92 (1.24–2.96)	0.01
Witnessed OHCA vs. non-witnessed OHCA	6.30 (4.73–8.40)	<0.001	2.65 (1.94–3.61)	<0.001
Shockable rhythm vs. non-shockable rhythm	10.36 (8.10–13.30)	<0.001	7.04 (5.33–9.28)	<0.001
OHCA during pandemic waves vs. intervals between pandemic waves	0.69 (0.55–0.85)	0.004	0.62 (0.48–0.79)	0.002

Abbreviation: OHCA = out-of-hospital cardiac arrest.

**Table 7:** Logistic regression analysis of survival to hospital admission in the 2021 patients who received CPR.

Variables	Unadjusted		Adjusted	
	OR (95% CI)	*p*-Value	OR (95% CI)	*p*-Value
OHCA near buildings vs. home and others	2.37 (1.62–3.47)	<0.001	2.05 (1.09–3.86)	0.063
Time to intervention	0.96 (0.94–0.98)	0.004	0.97 (0.94–1.01)	0.193
CPR by bystanders vs. no CPR by bystanders	1.70 (1.35–2.13)	<0.001	0.89 (0.58–1.37)	0.654
Phone-guided CPR vs. non-phone-guided CPR	1.50 (1.18–2.78)	0.005	0.84 (0.55–1.30)	0.516
Shock by bystander vs. shock by EMS	3.11 (1.42–6.83)	0.017	3.14 (1.34–7.36)	0.028

Abbreviations: EMS = emergency medical system; CPR = cardiopulmonary resuscitation; OHCA = out-of-hospital cardiac arrest.

## Data Availability

The data presented in this study are available upon reasonable request from the authors. The data are not publicly available due to privacy and ethical reasons.

## References

[1] Gräsner J.T., Wnent J., Herlitz J., Perkins G.D., Lefering R., Tjelmeland I., Koster R.W., Masterson S., Rossell-Ortiz F., Maurer H. (2020). Survival after out-of-hospital cardiac arrest in Europe—Results of the EuReCa TWO study. Resuscitation.

[2] Nolan J. (2005). European Resuscitation Council. European Resuscitation Council guidelines for resuscitation 2005. Section 1. Introduction. Resuscitation.

[3] Zeppenfeld K., Tfelt-Hansen J., de Riva M., Winkel B.G., Behr E.R., Blom N.A., Charron P., Corrado D., Dagres N., de Chillou C. (2022). 2022 ESC Guidelines for the management of patients with ventricular arrhythmias and the prevention of sudden cardiac death. Eur. Heart J..

[4] Scquizzato T., Gamberini L., D’Arrigo S., Galazzi A., Babini G., Losiggio R., Imbriaco G., Fumagalli F., Cucino A., Landoni G. (2022). Incidence, characteristics, and outcome of out-of-hospital cardiac arrest in Italy: A systematic review and meta-analysis. Resusc. Plus.

[5] Tjelmeland I.B.M., Alm-Kruse K., Grasner J.T., Isern C.B., Jakisch B., Kramer-Johansen J., Renzing N., Wnent J., Seewald S. (2022). Importance of reporting survival as incidence: A cross-sectional comparative study on out-of-hospital cardiac arrest registry data from Germany and Norway. BMJ Open.

[6] Tjelmeland I.B.M., Wnent J., Masterson S., Kramer-Johansen J., Ong M.E.H., Smith K., Skogvoll E., Lefering R., Lim S.L., Liu N. (2023). Did lockdown influence bystanders’ willingness to perform cardiopulmonary resuscitation? A worldwide registry-based perspective. Resuscitation.

[7] Baldi E., Primi R., Bendotti S., Currao A., Compagnoni S., Gentile F.R., Sechi G.M., Mare C., Palo A., Contri E. (2021). Relationship between out-of-hospital cardiac arrests and COVID-19 during the first and second pandemic wave. The importance of monitoring COVID-19 incidence. PLoS ONE.

[8] Perkins G.D., Jacobs I.G., Nadkarni V.M., Berg R.A., Bhanji F., Biarent D., Bossaert L.L., Brett S.J., Chamberlain D., de Caen A.R. (2015). Cardiac arrest and cardiopulmonary resuscitation outcome reports: Update of the Utstein Resuscitation Registry Templates for Out-of-Hospital Cardiac Arrest: A statement for healthcare professionals from a task force of the International Liaison Committee on Resuscitation (American Heart Association, European Resuscitation Council, Australian and New Zealand Council on Resuscitation, Heart and Stroke Foundation of Canada, InterAmerican Heart Foundation, Resuscitation Council of Southern Africa, Resuscitation Council of Asia); and the American Heart Association Emergency Cardiovascular Care Committee and the Council on Cardiopulmonary, Critical Care, Perioperative and Resuscitation. Circulation.

[9] Mappa Defibrillatore Automatico Esterno Lombardia. https://www.areu.lombardia.it/web/home/mappa-dae-lombardia.

[10] Boriani G., Guerra F., De Ponti R., D’Onofrio A., Accogli M., Bertini M., Bisignani G., Forleo G.B., Landolina M., Lavalle C. (2023). Five waves of COVID-19 pandemic in Italy: Results of a national survey evaluating the impact on activities related to arrhythmias, pacing, and electrophysiology promoted by AIAC (Italian Association of Arrhythmology and Cardiac Pacing). Intern. Emerg. Med..

[11] Park J.H., Song K.J., Do Shin S., Hong K.J. (2023). The impact of COVID-19 pandemic on out-of-hospital cardiac arrest system-of-care: Which survival chain factor contributed the most?. Am. J. Emerg. Med..

[12] Baldi E., Sechi G.M., Mare C., Canevari F., Brancaglione A., Primi R., Klersy C., Palo A., Contri E., Ronchi V. (2020). Out-of-Hospital Cardiac Arrest during the Covid-19 Outbreak in Italy. N. Engl. J. Med..

[13] Baldi E., Sechi G.M., Mare C., Canevari F., Brancaglione A., Primi R., Klersy C., Palo A., Contri E., Ronchi V. (2020). COVID-19 kills at home: The close relationship between the epidemic and the increase of out-of-hospital cardiac arrests. Eur. Heart J..

[14] Husain A.A., Rai U., Sarkar A.K., Chandrasekhar V., Hashmi M.F. (2023). Out-of-Hospital Cardiac Arrest during the COVID-19 Pandemic: A Systematic Review. Healthcare.

[15] Fordyce C.B., Hansen C.M., Kragholm K., Dupre M.E., Jollis J.G., Roettig M.L., Becker L.B., Hansen S.M., Hinohara T.T., Corbett C.C. (2017). Association of Public Health Initiatives With Outcomes for Out-of-Hospital Cardiac Arrest at Home and in Public Locations. JAMA Cardiol..

[16] Karlsson L., Hansen C.M., Vourakis C., Sun C.L., Rajan S., Søndergaard K.B., Andelius L., Lippert F., Gislason G.H., Chan T.C. (2020). Improving bystander defibrillation in out-of-hospital cardiac arrests at home. Eur. Heart J. Acute Cardiovasc. Care.

[17] Gamberini L., Mazzoli C.A., Allegri D., Scquizzato T., Baroncini S., Guarnera M., Tartaglione M., Chiarini V., Picoco C., Semeraro F. (2022). Factors influencing prehospital physicians’ decisions to initiate advanced resuscitation for asystolic out-of-hospital cardiac arrest patients. Resuscitation.

[18] Oving I., de Graaf C., Masterson S., Koster R.W., Zwinderman A.H., Stieglis R., AliHodzic H., Baldi E., Betz S., Cimpoesu D. (2020). European first responder systems and differences in return of spontaneous circulation and survival after out-of-hospital cardiac arrest: A study of registry cohorts. Lancet Reg. Health Eur..

[19] Zijlstra J.A., Koster R.W., Blom M.T., Lippert F.K., Svensson L., Herlitz J., Kramer-Johansen J., Ringh M., Rosenqvist M., Palsgaard Møller T. (2018). Different defibrillation strategies in survivors after out-of-hospital cardiac arrest. Heart.

[20] Hubar I., Fischer M., Monaco T., Gräsner J.T., Westenfeld R., Bernhard M. (2023). Development of the epidemiology and outcomes of out-of-hospital cardiac arrest using data from the German Resuscitation Register over a 15-year period (EpiCPR study). Resuscitation.

[21] Kitamura T., Kiyohara K., Sakai T., Matsuyama T., Hatakeyama T., Shimamoto T., Izawa J., Fujii T., Nishiyama C., Kawamura T. (2016). Public-Access Defibrillation and Out-of-Hospital Cardiac Arrest in Japan. N. Engl. J. Med..

[22] Pollack R.A., Brown S.P., Rea T., Aufderheide T., Barbic D., Buick J.E., Christenson J., Idris A.H., Jasti J., Kampp M. (2018). Impact of Bystander Automated External Defibrillator Use on Survival and Functional Outcomes in Shockable Observed Public Cardiac Arrests. Circulation.

[23] Karam N., Narayanan K., Bougouin W., Benameur N., Beganton F., Jost D., Lamhaut L., Perier M.C., Cariou A., Celermajer D.S. (2017). Major regional differences in Automated External Defibrillator placement and Basic Life Support training in France: Further needs for coordinated implementation. Resuscitation.

[24] Mpotos N., De Wever B., Cleymans N., Raemaekers J., Loeys T., Herregods L., Valcke M., Monsieurs K.G. (2014). Repetitive sessions of formative self-testing to refresh CPR skills: A randomised non-inferiority trial. Resuscitation.

[25] Schroeder D.C., Semeraro F., Greif R., Bray J., Morley P., Parr M., Kondo Nakagawa N., Iwami T., Finke S.R., Malta Hansen C. (2023). KIDS SAVE LIVES: Basic Life Support Education for Schoolchildren: A Narrative Review and Scientific Statement From the International Liaison Committee on Resuscitation. Circulation.

[26] Ringh M., Rosenqvist M., Hollenberg J., Jonsson M., Fredman D., Nordberg P., Järnbert-Pettersson H., Hasselqvist-Ax I., Riva G., Svensson L. (2015). Mobile-phone dispatch of laypersons for CPR in out-of-hospital cardiac arrest. N. Engl. J. Med..

[27] Stroop R., Kerner T., Strickmann B., Hensel M. (2020). Mobile phone-based alerting of CPR-trained volunteers simultaneously with the ambulance can reduce the resuscitation-free interval and improve outcome after out-of-hospital cardiac arrest: A German, population-based cohort study. Resuscitation.

[28] Lee S.Y., Shin S.D., Lee Y.J., Song K.J., Hong K.J., Ro Y.S., Lee E.J., Kong S.Y. (2019). Text message alert system and resuscitation outcomes after out-of-hospital cardiac arrest: A before-and-after population-based study. Resuscitation.

[29] Andelius L., Malta Hansen C., Lippert F.K., Karlsson L., Torp-Pedersen C., Kjær Ersbøll A., Køber L., Collatz Christensen H., Blomberg S.N., Gislason G.H. (2020). Smartphone Activation of Citizen Responders to Facilitate Defibrillation in Out-of-Hospital Cardiac Arrest. J. Am. Coll. Cardiol..

[30] Scquizzato T., Belloni O., Semeraro F., Greif R., Metelmann C., Landoni G., Zangrillo A. (2022). Dispatching citizens as first responders to out-of-hospital cardiac arrests: A systematic review and meta-analysis. Eur. J. Emerg. Med..

[31] Scapigliati A., Semeraro F., Di Marco S., Ristagno G., Italian Resuscitation Council Executive Committee (2021). The new Italian law “A systems saving lives” the first European former application of ERC 2021 guidelines. Resuscitation.

